# Supplementary material for: Regulation of Epigenetic Modifications in the Placenta during Preeclampsia: PPARγ Influences H3K4me3 and H3K9ac in Extravillous Trophoblast Cells
Source: Int J Mol Sci. 2021 Nov 18;22(22):12469. doi: 10.3390/ijms222212469 (PMC8622744; doi:10.3390/ijms222212469)
Supplement: Supplementary file 1 [file ijms-22-12469-s001.zip › ijms-1455846-supplementary.pdf]

Supplementary Material

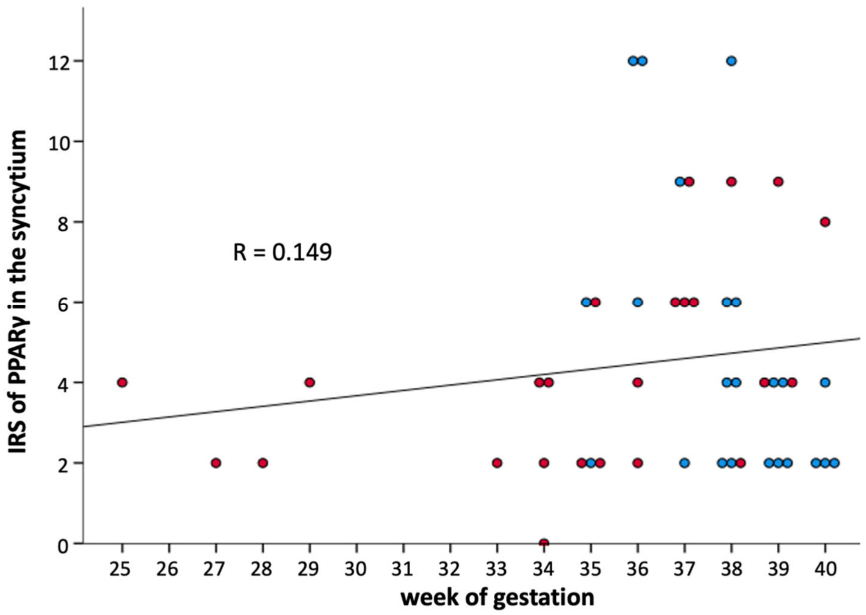

Figure S1. Visualization of histone expression in relation to the weeks of gestation. The regression line represents no significant correlation ($p = 0.316$). Linear regression was used to analyze the effect of the week of gestation on the IRS of PPARγ in the syncytium.

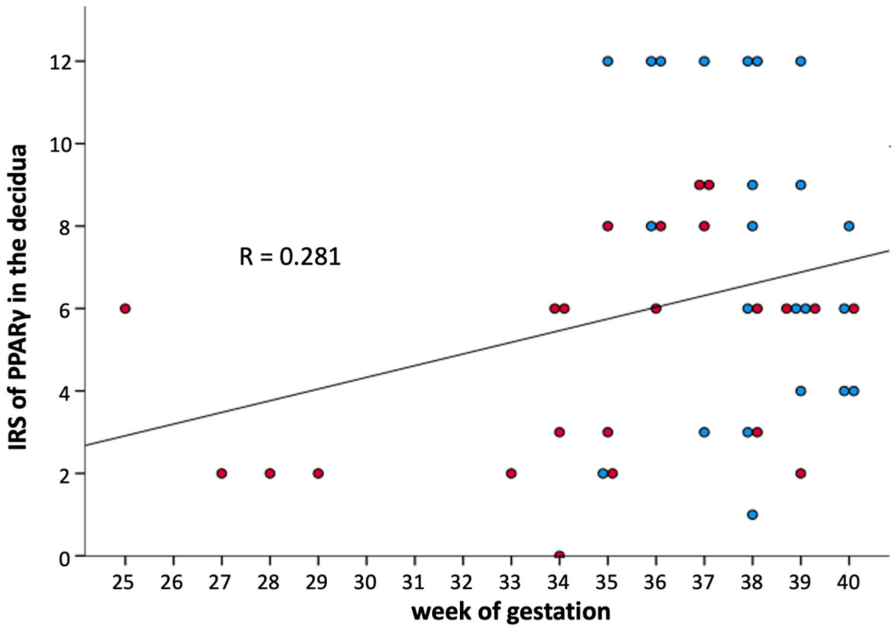

Figure S2. Visualization of histone expression in relation to the weeks of gestation. The regression line represents no significant correlation ($p = 0.058$). Linear regression was used to analyze the effect of the week of gestation on the IRS of PPARγ in the decidua.

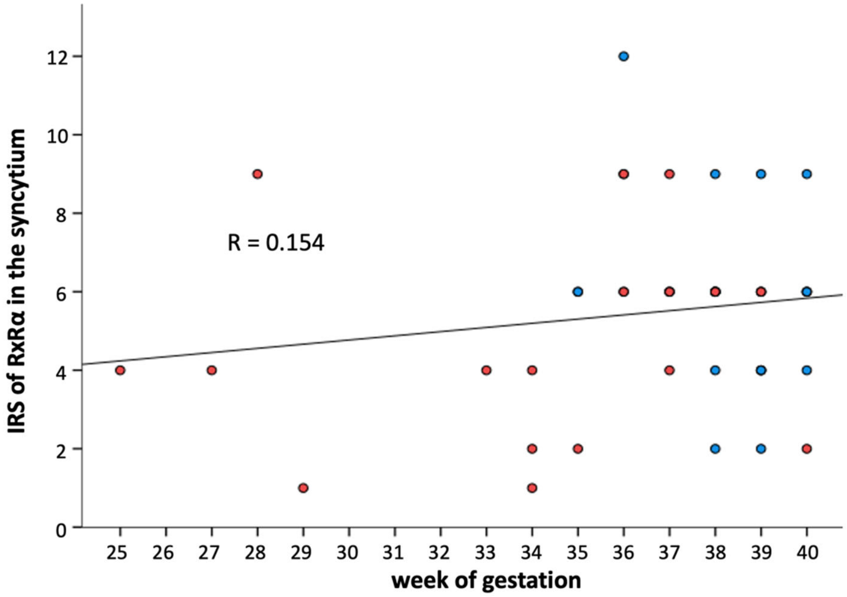

Figure S3. Visualization of histone expression in relation to the weeks of gestation. The regression line represents no significant correlation ($p = 0.326$). Linear regression was used to analyze the effect of the week of gestation on the IRS of RxRα in the syncytium.

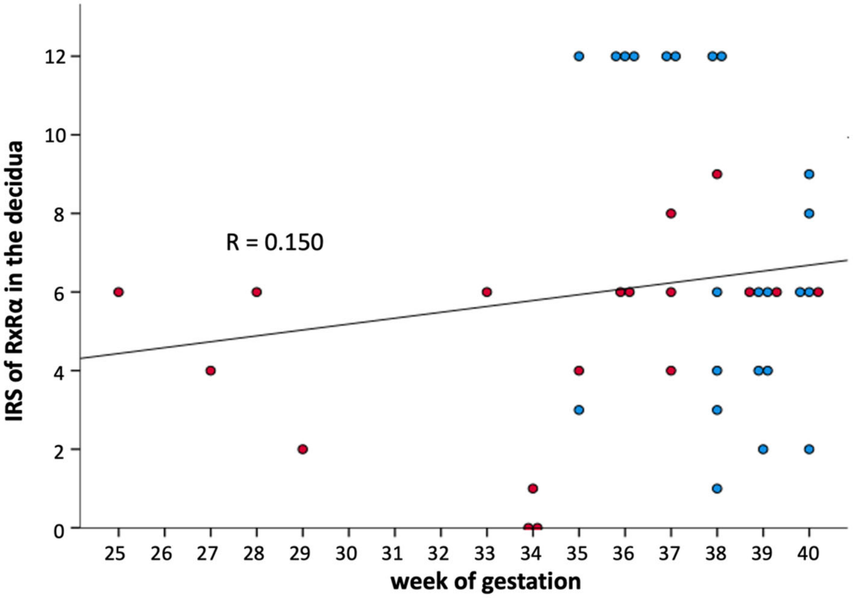

Figure S4. Visualization of histone expression in relation to the weeks of gestation. The regression line represents no significant correlation ($p = 0.350$). Linear regression was used to analyze the effect of the week of gestation on the IRS of RxRα in the decidua.

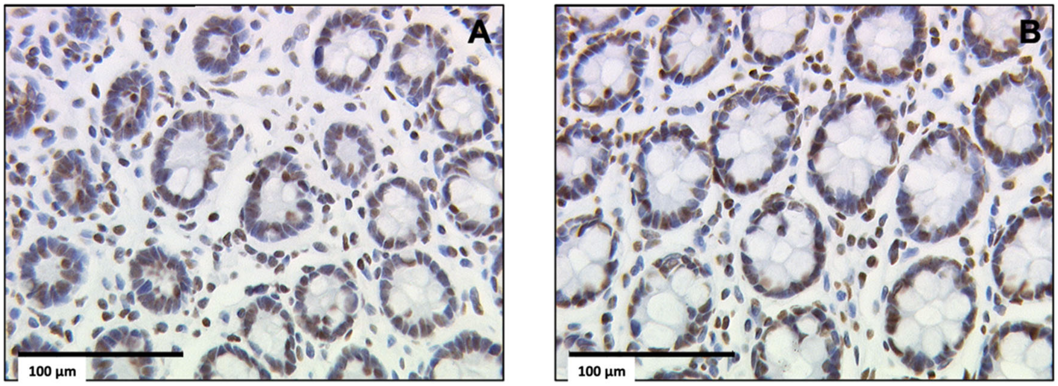

Figure S5. Positive control for PPARγ and RxRα. Representative immunohistochemistry images of positive control stainings. (**A**) expression of PPARγ in human colon tissue, scale bar: 100 µm. (**B**) expression of RxRα in human colon tissue, scale bar: 100 µm.

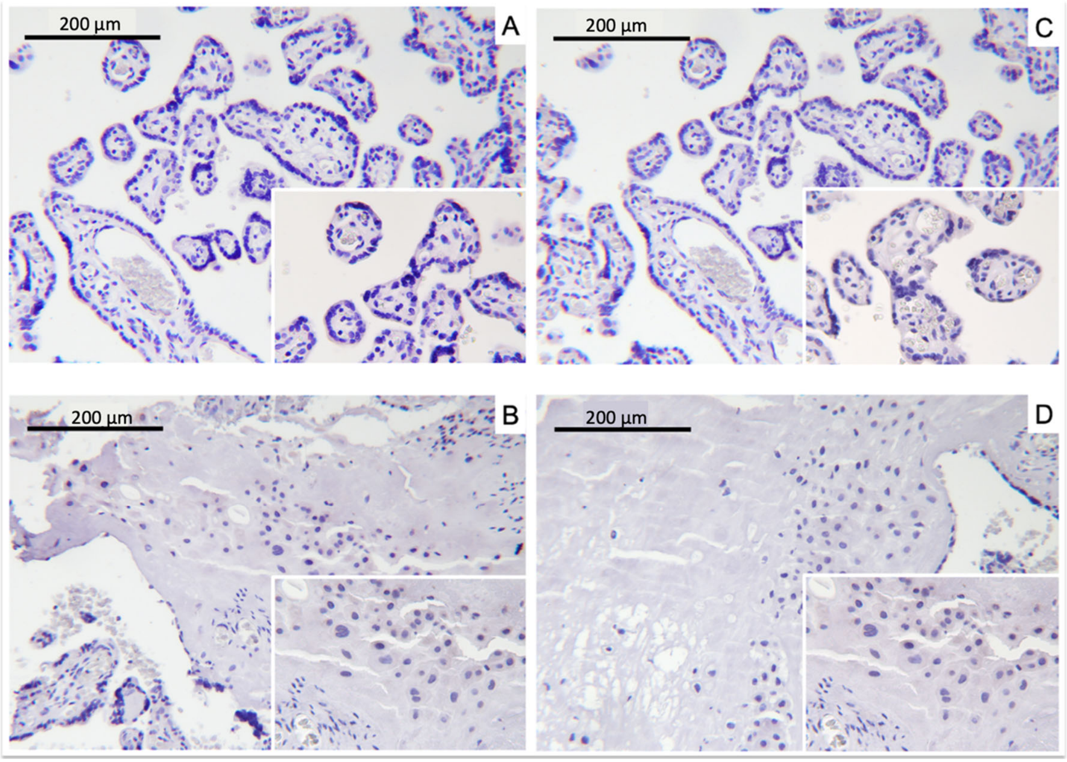

Figure S6. Negative control for PPARγ (**A**, **B**) and RxRα (**C**, **D**). Representative immunohistochemistry images of negative control stainings of placental tissue. (**A**, **C**) villous trophoblast; (**B**, **D**) decidua. scale bar: 200 µm.

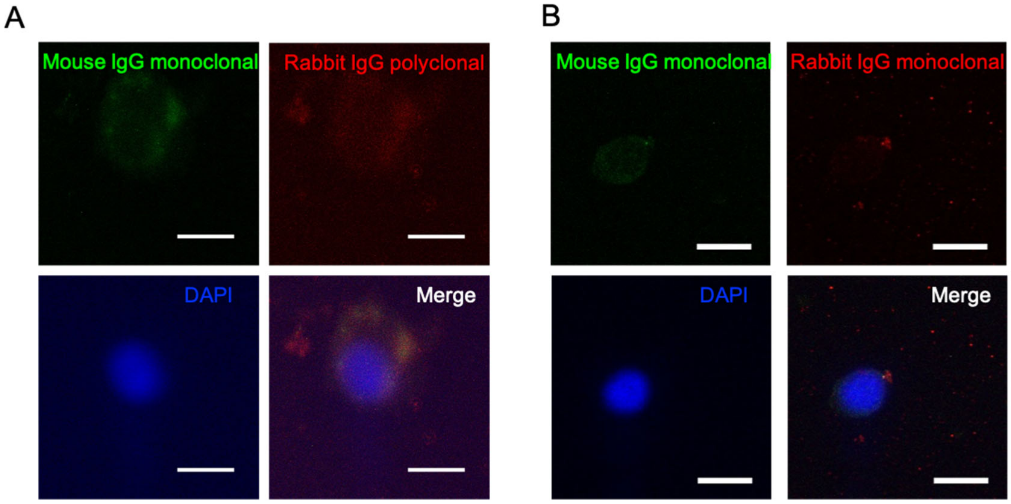

Figure S7. IF double staining: Negative controls for PPARγ, H3K9ac and H3K4me3, representative pictures. Respective Isotype IgG for PPARγ in green and for H3K9me3 in red (**A**), respective Isotype IgG for PPAR γ and H3K9ac in red (**B**). Scale bar 10 μm.

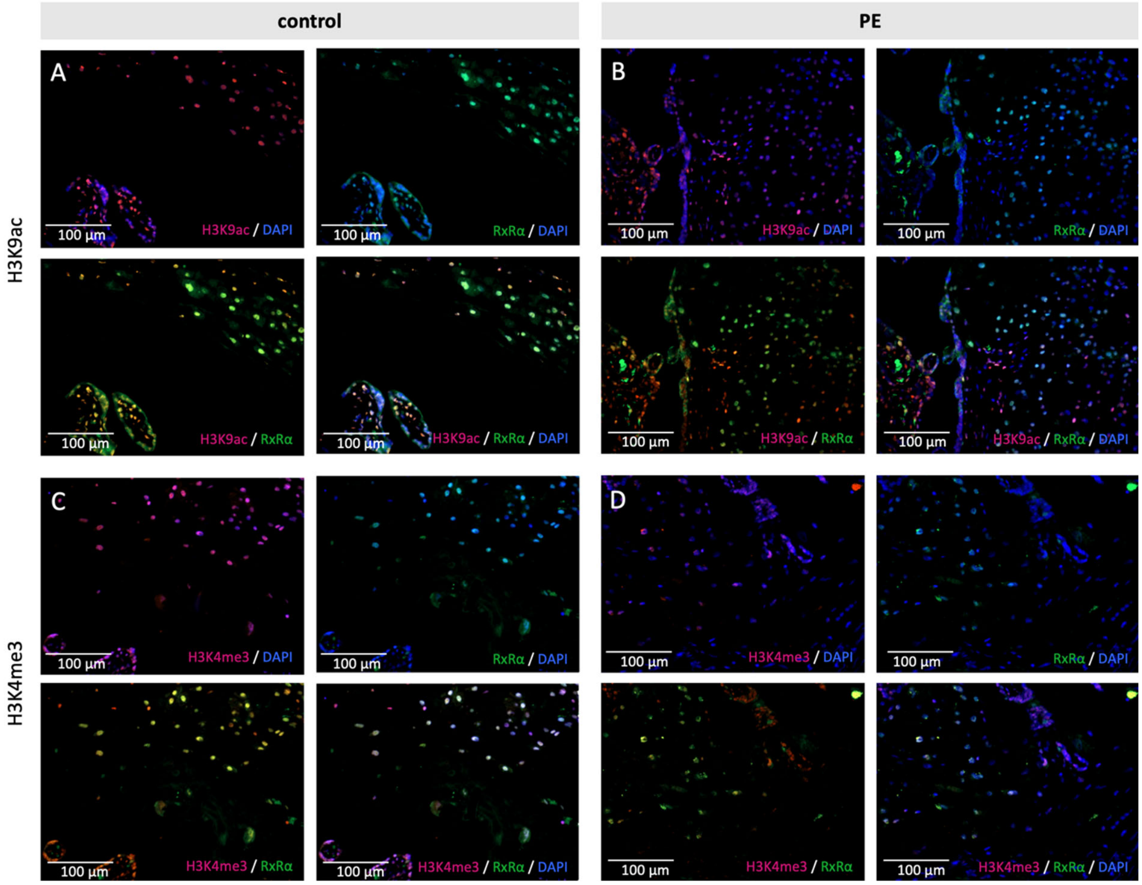

Figure S8. Examples of staining results of the immunofluorescence of RxRα and H3K9ac (**A**, **B**) and H3K4me3 (**C**, **D**), in control and PE placentas. Single immunofluorescence staining of H3K9ac and H3K4me3 (pink). Single immunofluorescence staining of RxRα (green). Double immunofluorescence staining of H3K9ac (**A**, **B**) and H3K4me3 (**C**, **D**) (red) and RxRα (green). DAPI as nucleus staining (blue). Scale bar 100 μm.

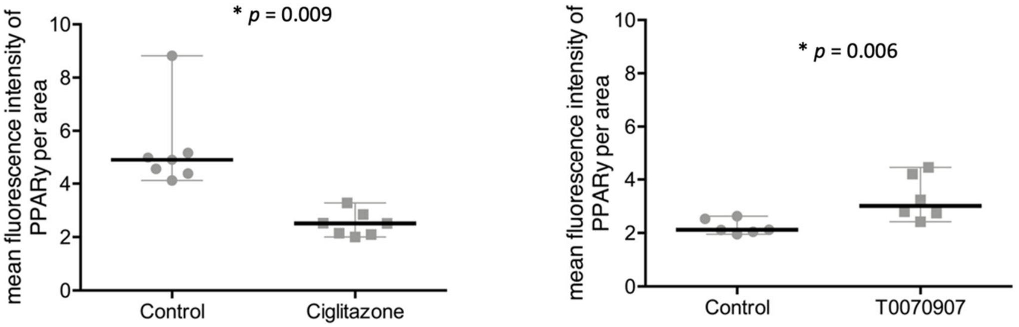

Figure S9 Mean fluorescence intensity of PPARγ in primary isolated EVTs after incubation with Ciglitazone (**A**) (20 mM) and T0070907 (**B**, **D**) (50 mM) mean fluorescence intensity ± SD; The respective *p*-value indicates, if the intensity of untreated control cells and cells incubated with Ciglitazone or T0070907 differ significantly and was calculated using the Mann-Whitney-U-Test.

Table S1. Gestational age and sex of the babies of healthy and PE affected pregnancies. Missing information are presented as "-".

Controls				PE					
Week of Gestation	Sex of the Baby	Weight of the Baby (g)	APGAR	Week of Gestation	Sex of the Baby	Weight of the Baby (g)	APGAR	Proteinuria (mg/dL)	Blood Pressure (mmHg)
40 + 3	male	-	-	40 + 0	male	3110	10/10/10	33.7	190/111
40 + 2	female	-	-	39 + 2	male	3750	4/7/9	82	143/88
40 + 2	female	-	-	39 + 2	male	3560	9/10/10	300	156/93
40 + 1	female	-	-	39 + 0	male	3260	10/10/10	300	170/100
40 + 0	female	3260	9/10/10	38 + 0	male	3030	10/10/10	-	-
39 + 6	male	-	-	38 + 0	female	2955	9/10/10	300	160/113
39 + 6	female	-	-	37 + 6	male	2530	8/10/10	-	157/84
39 + 1	male	3675	9/10/10	37 + 5	female	2830	8/10/10	72	165/95
39 + 1	male	-	-	37 + 3	male	2685	8/10/10	33.8	155/115
39 + 1	male	-	-	37 + 2	female	2750	9/10/10	-	-
38 + 4	male	2790	9/10/10	36 + 1	female	2650	3/9/10	587	152/77
38 + 4	female	-	-	36 + 1	female	2220	5/8/10	587	152/78
38 + 4	male	2650	7/8/9	35 + 3	female	2500	9/10/10	191	166/88
38 + 3	female	2875	9/10/10	35 + 0	female	2330	8/10/10	221	170/83
38 + 3	female	-	-	35 + 0	female	1555	8/10/10	-	180/100
38 + 2	female	3675	9/10/10	34 + 6	-	-	-	-	-
38 + 1	female	3310	9/10/10	34 + 1	male	1704	9/10/10	-	-
38 + 0	male	-	-	34 + 1	female	1735	8/10/10	417	180/120
37 + 0	female	2580	9/10/10	34 +1	male	2000	7/9/10	300	190/100
37 + 0	male	2430	9/10/10	33 + 5	female	1770	7/9/9	35.2	149/101
36 + 5	male	3130	8/9/10	29 + 5	-	-	-	-	-
36 + 0	female	2550	9/10/10	28 + 6	female	930	8/9/10	140	155/97
36 + 0	female	2260	9/10/10	27 + 1	male	680	8/8/8	39.8	170/110
35 + 6	female	2640	9/10/10	25 + 6	male	570	4/7/9	-	-
35 + 1	female	-	-	-	-	-	-	-	-
				-	-	-	-	-	-

Table S2. Exemplary images of staining intensities scored by performing the IRS.

	No staining = 0	Weak staining = 1	Moderate staining = 2	Strong staining = 3
PPARγ	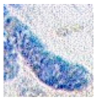	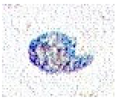	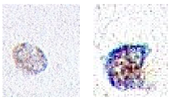	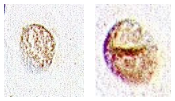
RxRα	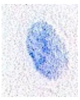	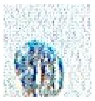	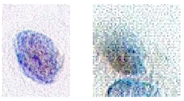	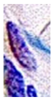
